# Orthodontic Extrusion of a Traumatically Intruded and Subsequently Ankylosed Incisor Following Surgical Luxation: A Case Report

**DOI:** 10.1155/crpe/2564793

**Published:** 2026-07-02

**Authors:** Kun Li, Deming Yu, Mengchao Chi, Mingna Tang

**Affiliations:** ^1^ Fushan Division, The Affiliated Yantai Stomatological Hospital, Binzhou Medical University, Yantai, China, bzmc.edu.cn; ^2^ Wolong Division, The Affiliated Yantai Stomatological Hospital, Binzhou Medical University, Yantai, China, bzmc.edu.cn; ^3^ Department of Pediatric Dentistry, The Affiliated Yantai Stomatological Hospital, Binzhou Medical University, Yantai, China, bzmc.edu.cn

**Keywords:** intrusive luxation, orthodontic extrusion, surgical luxation, traumatic ankylosis

## Abstract

**Background:**

The self‐healing process following intrusive luxation of a tooth is often associated with serious complications, such as ankylosis. Once traumatic ankylosis is confirmed, spontaneous re‐eruption of the intruded tooth is unlikely, and timely intervention is crucial to facilitate its repositioning.

**Case Report:**

This case report details the systematic management of an adolescent patient with a traumatically intruded incisor. Ankylosis was diagnosed after the tooth showed no spontaneous re‐eruption during monitoring and failed to respond to orthodontic traction. Surgical luxation was promptly performed, and the ankylosed incisor was effectively guided into position following subsequent orthodontic extrusion. Throughout the follow‐up period, the treatment outcomes remained stable, with no adverse sequelae observed.

**Conclusion:**

This case report demonstrates that surgical luxation combined with orthodontic extrusion can be one of the effective approaches for realigning traumatically ankylosed permanent teeth.

## 1. Introduction

Dental trauma is among the most common oral conditions in adolescents, second only to caries. In regions with a sharp decline in caries prevalence, it may even become the primary concern. Among traumatic dental injuries, intrusive luxation is considered one of the most severe types, accounting for approximately 0.3%–2% of all dental injuries in the permanent dentition [1]. It is characterized by apical displacement of the tooth into the alveolar bone and is frequently associated with extensive damage to the periodontal ligament and neurovascular supply. Ankylosis may occur when the root surface (cementum or dentin) becomes directly fused to the surrounding alveolar bone, further complicating treatment and adversely affecting the long‐term prognosis.

Although the management of intrusive luxation in permanent teeth remains clinically challenging, current treatment strategies are guided by evidence‐based recommendations. The 2020 guidelines from the International Association of Dental Traumatology (IADT) provide structured management protocols based on the degree of intrusion and the stage of root development [2]. In general, spontaneous re‐eruption is recommended for immature permanent teeth, as well as for mature teeth with mild intrusion. In contrast, active repositioning (either orthodontic or surgical) is indicated for mature teeth with moderate to severe intrusion. A systematic review concluded that, irrespective of the intrusion severity, spontaneous re‐eruption carries the lowest complication rate [3]. Despite these established recommendations, variations in clinical practice have been reported in the literature [4–6]. In patients aged 12–17 years with fully developed roots, spontaneous re‐eruption has also been reported [7]; however, the outcome is less predictable and requires close monitoring and timely intervention when indicated.

A substantial challenge for clinicians is the frequent occurrence of posttraumatic ankylosis in permanent incisors. An ankylosed incisor cannot re‐erupt spontaneously, and orthodontic repositioning typically fails, often resulting in unintended movement of the anchorage teeth. The diagnosis of tooth ankylosis is primarily based on a distinct metallic sound on percussion, the absence of physiological mobility, and radiographic loss of the periodontal ligament space. In addition, the presence of infraposition of the affected tooth relative to adjacent teeth may serve as an important clinical indicator of ankylosis. Nevertheless, in clinical practice, ankylosis is frequently underdiagnosed due to challenges in distinguishing the percussion sounds. Additionally, when the ankylosed region is small or confined to the labial or lingual surfaces, it may not be visible on two‐dimensional radiographs. Ankylosis can only be definitively diagnosed when the affected tooth exhibits no movement despite the application of orthodontic force [8].

Surgical luxation involves deliberately mobilizing an ankylosed tooth within its socket to disrupt pathological ankylosis, thereby facilitating subsequent orthodontic realignment [9]. Combined surgical luxation and orthodontic treatment present a potential approach for managing infrapositioned ankylosed incisors. This article describes a case of an adolescent who attained favorable treatment outcomes through this technique.

## 2. Case Report

An 11‐year‐old patient sustained dental injuries in an accident and sought treatment two hours posttrauma. Prior to undergoing a dental examination, the patient underwent a thorough evaluation in the emergency department, where traumatic brain injury was ruled out. Clinical examination revealed a middle‐third crown fracture in Tooth 11 and a cervical‐third fracture in Tooth 21, both with pulp exposure, with the fractured crown segments completely lost. Tooth 21 exhibited an intrusion depth of approximately 5 mm, with only a minimal portion visible intraorally and the distal end of the fracture line located approximately 2 mm subgingivally. Tooth 22 was displaced lingually, resulting in premature occlusal contact with its antagonist and demonstrating Grade I mobility. Gingival erythema, swelling, and ecchymosis were observed around Teeth 11, 21, and 22. Cold testing revealed diminished responses in these teeth compared to the controls. The periapical radiograph revealed that Tooth 21 was positioned infraocclusally relative to the adjacent teeth (Figure 1). The roots of Teeth 11, 21, and 22 were nearly fully developed, with no signs of root fractures observed. The periodontal ligament space of Tooth 11 appeared widened, while that of Tooth 21 was absent. There was no clinical or radiographic evidence of alveolar fracture.

**FIGURE 1:**
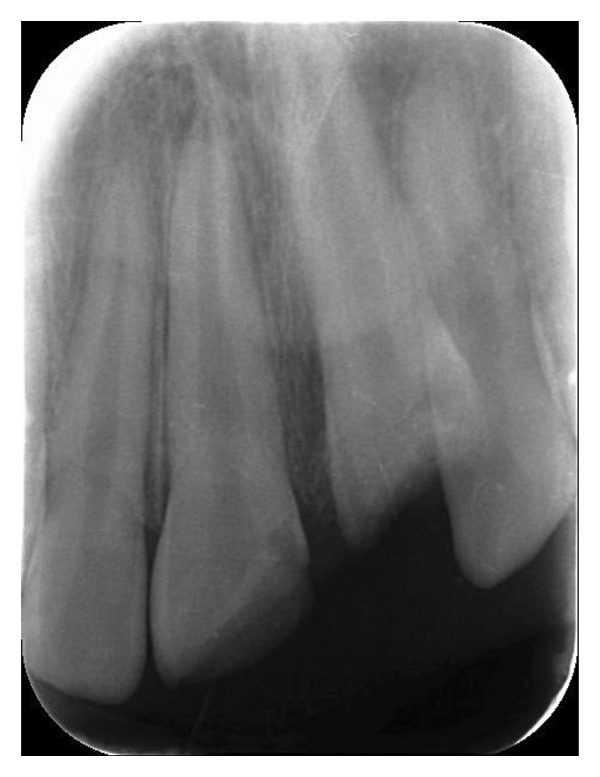
Pretreatment periapical radiograph obtained immediately postinjury.

The initial diagnosis included a complicated crown fracture of Tooth 11, an intrusive luxation with a complicated crown fracture of Tooth 21, and lateral luxation of Tooth 22.

After a comprehensive evaluation of the patient’s condition and interdisciplinary consultation, a preliminary treatment plan was proposed. The plan included a pulpotomy for Tooth 11 with subsequent monitoring of pulp vitality, and root canal therapy to be considered if necessary; root canal therapy for Tooth 21 without immediate repositioning, with close observation of its potential spontaneous re‐eruption and further intervention if indicated; immediate manual repositioning of Tooth 22; and placement of a wire splint to stabilize the affected dentition and maintain space for Tooth 21. It is noteworthy that the management strategy for Tooth 21 in this case deviated from the recommendations of the IADT guidelines, which advocate active repositioning for mature teeth with moderate to severe intrusion. This deviation from the standard protocol was made following careful consideration of several patient‐specific factors. These included the patient’s young age and the fact that root development had only recently been completed. In addition, previous reports have suggested that spontaneous re‐eruption may still occur in selected cases of intruded teeth with complete root formation. Furthermore, immediate repositioning procedures may introduce additional trauma to the periodontal ligament and surrounding tissues. Therefore, an initial period of close observation was considered appropriate to minimize the risk of secondary injury, with readiness to proceed to active intervention if spontaneous re‐eruption did not occur.

The treatment plan was presented to the patient’s parents in detail, including an explanation of the diagnosis, proposed procedures, potential risks, complications, and long‐term prognosis. Informed consent was subsequently obtained and documented after confirming their full understanding and agreement to proceed.

Following local anesthesia, the pulp exposure site of Tooth 11 was carefully enlarged to ensure complete visualization of the pulp chamber. Coronal pulp amputation was performed using a sterile round bur under copious saline irrigation. A double‐layer pulp capping technique was then applied, with iRoot BP Plus cement (Innovative Bioceramix Inc., Vancouver, Canada) placed directly over the pulp tissue, followed by a layer of light‐cured calcium hydroxide cement. The tooth was subsequently restored with a temporary glass ionomer cement filling. For Tooth 21, pulpectomy was carried out with meticulous canal debridement and copious irrigation, followed by the placement of a calcium hydroxide intracanal dressing and temporary sealing with glass ionomer cement. Tooth 22 was manually repositioned to its correct anatomical position, followed by careful occlusal adjustment. Finally, a passive wire‐composite splint was bonded to Teeth 13, 12, 11, 22, and 23 to stabilize the affected teeth and preserve space for the potential spontaneous re‐eruption of Tooth 21 (Figure 2).

**FIGURE 2:**
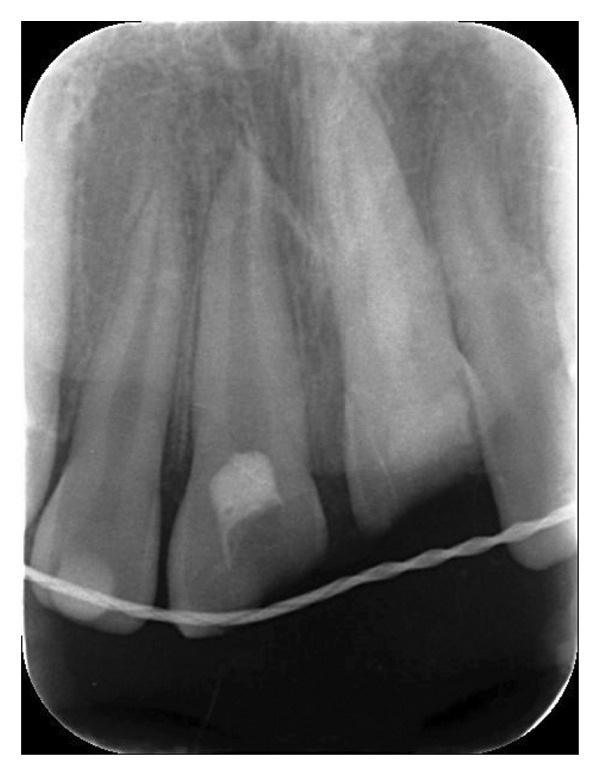
Periapical radiograph obtained immediately following emergency management. Tooth 11 underwent pulpotomy. Tooth 21 received pulpectomy followed by placement of an intracanal calcium hydroxide dressing. Tooth 22 was manually repositioned into its anatomical position. A passive wire‐composite splint was bonded from Tooth 13 to 23.

Postoperative instructions were thoroughly communicated to the patient’s parents, including guidance on oral care and activity restrictions. The importance of regular follow‐up visits for clinical and radiographic monitoring was emphasized.

At the two‐week follow‐up, clinical examination showed that mobility of Tooth 22 had resolved, and the splint was carefully removed. However, Tooth 21 remained in its intruded position with no evidence of spontaneous re‐eruption (Figure 3). No other findings required immediate intervention. The patient’s parents were updated on the current progress, with clear explanation that the treatment was still ongoing. The need for continued regular follow‐up was emphasized, and they were advised that if spontaneous re‐eruption of Tooth 21 did not occur within an appropriate observation period, additional measures would be necessary.

**FIGURE 3:**
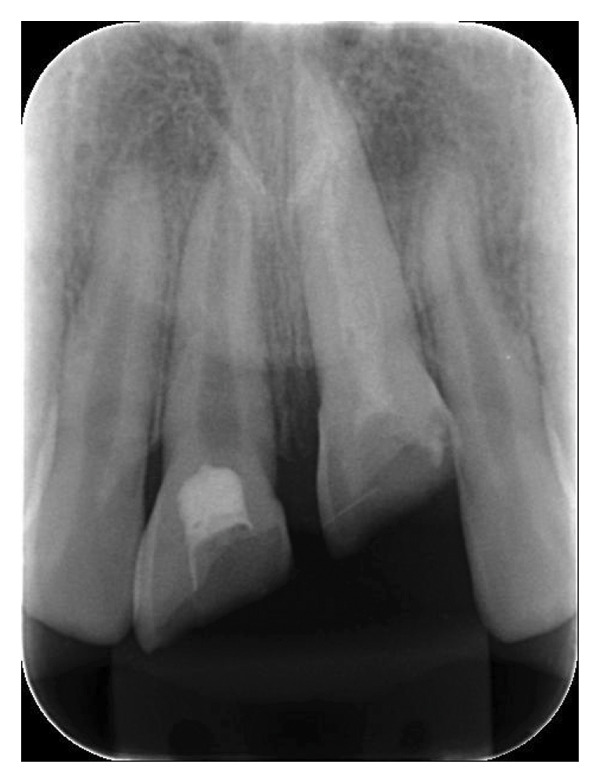
Periapical radiograph taken at 2 weeks postinjury. Mobility of Tooth 22 had resolved, and the splint was removed. Tooth 21 remained in its intruded position without signs of spontaneous re‐eruption.

At the 3‐month follow‐up, Tooth 21 remained intruded, prompting the decision to initiate orthodontic traction using a bonded traction chain and a removable appliance. Despite three months of traction, no significant movement was observed (Figure 4). Brackets were bonded from Tooth 14 to 24, followed by surgical luxation to gently mobilize Tooth 21 (Figure 5). A 0.012‐inch (in) nickel–titanium (NiTi) archwire was then engaged to initiate orthodontic alignment. Within one month, Tooth 21 demonstrated significant extrusion. At this point, root canal therapy was completed for Tooth 21, and the contours of Teeth 11 and 21 were restored with composite resin. Subsequently, 0.014‐inch, 0.016‐inch, and 0.018‐inch NiTi archwires were sequentially placed to achieve further alignment.

**FIGURE 4:**
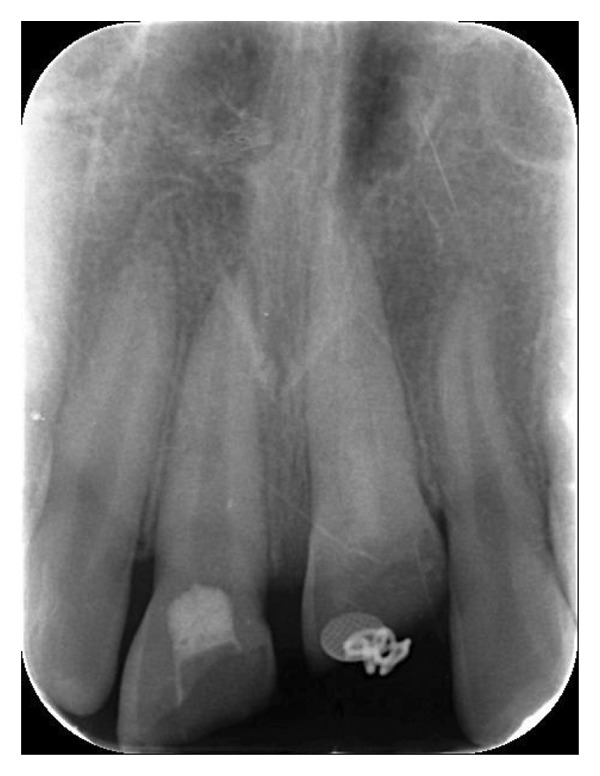
Periapical radiograph taken at 6 months postinjury (after three months of orthodontic traction). A traction chain was bonded to Tooth 21, and orthodontic traction was applied in conjunction with a removable appliance. No significant movement was observed after three months of traction.

**FIGURE 5:**
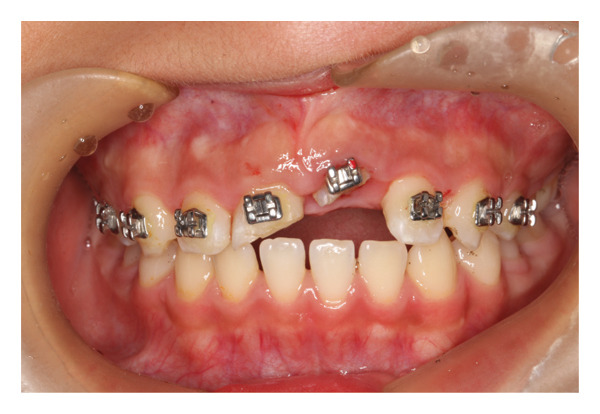
Intraoral photograph taken at approximately 6 months postinjury, following bracket bonding and surgical luxation of Tooth 21.

At the nine‐month follow‐up after the trauma, Tooth 21 was fully aligned. However, the patient reported recent biting discomfort in Tooth 11. Clinical examination revealed percussion tenderness, and a periapical radiograph showed an ill‐defined radiolucent area around the apex, indicating periapical inflammation (Figure 6). Given the history of previous pulpotomy on Tooth 11, root canal therapy was performed to address the progression of pulp necrosis. As the patient’s parents were satisfied with the treatment outcomes and declined further comprehensive orthodontic therapy for occlusal refinement, all brackets were removed, marking the completion of the overall treatment process.

**FIGURE 6:**
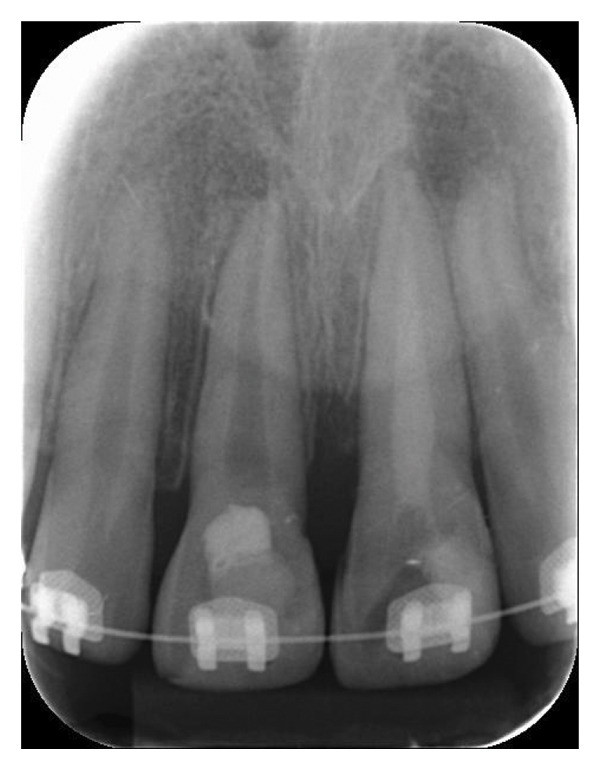
Periapical radiograph taken at 9 months postinjury, showing an ill‐defined radiolucent area around the apex of Tooth 11.

Intraoral photographs taken at the completion of the entire treatment revealed that Tooth 21 had been successfully repositioned, with appropriate restoration of the traumatic defects and alignment of the anterior teeth (Figure 7). However, sequelae of the previous intrusion remained evident, including an apically positioned gingival margin of Tooth 21 and recession of the interdental papilla between Teeth 11 and 21. The periapical radiograph taken at the completion of treatment revealed that the apex of Tooth 21 had nearly re‐established its position relative to that of Tooth 11 (Figure 8). Both teeth exhibited well‐obturated root canals, with resolution of the previously noted periapical radiolucency around Tooth 11. Nonetheless, an alveolar crest defect was observed between Teeth 11 and 21, along with mild apical resorption in both teeth.

**FIGURE 7:**
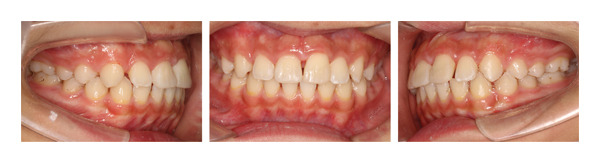
Intraoral photograph taken at the completion of treatment (approximately 9 months postinjury).

**FIGURE 8:**
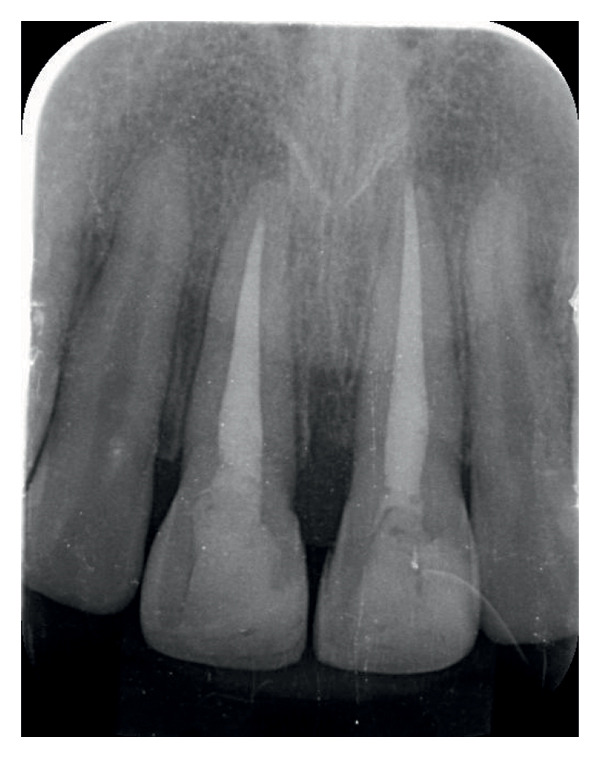
Periapical radiograph taken at the completion of treatment (approximately 9 months postinjury).

At the two‐year posttreatment follow‐up, intraoral photographs revealed slight positional changes in the previously aligned anterior teeth (Figure 9). Tooth 21 exhibited minor elongation, and the interdental papilla between Teeth 11 and 21 showed persistent recession, with the gingival margin of Tooth 21 remaining apically positioned. No additional significant sequelae or complications related to the initial trauma or its treatment were observed. The panoramic radiograph confirmed the stability of the treatment outcomes, showing no progression of pathological changes in the pulp, root, or alveolar bone of the affected anterior teeth (Figure 10).

**FIGURE 9:**
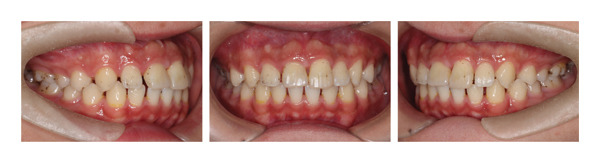
Intraoral photographs taken at 2 years of posttreatment follow‐up (approximately 2 years and 9 months postinjury).

**FIGURE 10:**
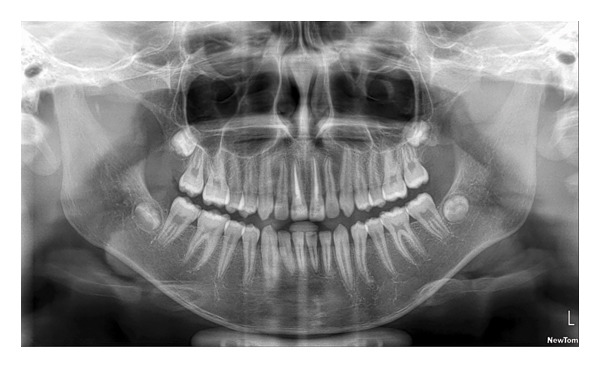
Panoramic radiograph taken at 2 years of posttreatment follow‐up (approximately 2 years and 9 months postinjury).

## 3. Discussion

Current recommendations from the IADT guidelines provide an evidence‐based framework for treatment selection based on the root development stage and the degree of intrusion. It should be emphasized that the initial emergency management of Tooth 21 in this case deviated from these recommendations, which advocate active repositioning for mature teeth with moderate to severe intrusion. Such a deviation may have been associated with the subsequent development of ankylosis occurring in an infrapositioned state, although a direct causal relationship cannot be definitively established. The initial decision to forgo immediate active repositioning of Tooth 21 was based on the assumption that a short observation period might allow periodontal ligament reorganization, potentially providing early signs of passive repositioning [10]. This conservative approach was also informed by previous reports suggesting that spontaneous re‐eruption may occur in selected cases, particularly when patient‐specific factors are taken into consideration [11]. The absence of spontaneous re‐eruption may be attributed to the extent of periodontal tissue disruption. The intruded incisor was wedged to such an extent that the root surface came into close contact with the alveolar bone. The preservation of the periodontal ligament in such a severe case was nearly inconceivable. The cementum was either sheared or torn from the root surface. The blood vessels in the periodontal complex were compressed, leading to ischemia. If these conditions persist, there is a high likelihood of healing complications, such as external replacement resorption, which may subsequently lead to ankylosis. However, a retrospective study on treatment outcomes revealed that replacement resorption occurred in 5% of teeth that were allowed to re‐erupt, compared to 29% in those treated with both surgical and orthodontic repositioning [12]. This difference was statistically significant, suggesting that active repositioning (either surgical or orthodontic) may be associated with an increased risk of replacement resorption. This finding further supports the decision to initially adopt a conservative observation approach for this patient.

Replacement resorption is rarely identifiable or predictable in the immediate or early posttraumatic phase. However, its incidence in intruded incisors is not negligible. In the absence of spontaneous re‐eruption within a reasonable observation period, replacement resorption and subsequent ankylosis should be highly suspected. Orthodontic repositioning is ineffective in cases of severe intrusion complicated by ankylosis [13]. In this case, Tooth 21 exhibited no positional change after three months of passive observation followed by three months of traction using a removable appliance, confirming a diagnosis of ankylosis.

Following confirmation of ankylosis, prompt and appropriate management is essential to mitigate its long‐term prognostic impact. Management options for ankylosed incisors are varied, but each carries inherent limitations. A conventional approach entails removal of the ankylosed tooth, followed by space rehabilitation with either a fixed dental prosthesis or an implant‐supported restoration. This approach is limited by the alveolar bone defect resulting from growth arrest due to ankylosis and additional bone loss during tooth extraction. Unmanaged bone defect may undermine esthetics and complicate treatment. Segmental osteotomy in conjunction with orthodontic traction enables movement of ankylosed teeth and has been widely reported [14, 15]. Corticotomy, involving targeted cortical incisions and subsequent orthodontic traction, offers another surgical option [16, 17]. However, due to growth deficiency of the ankylosed alveolar segment, the risk of infraocclusion recurrence remains a key consideration prior to these surgical interventions. Orthodontically assisted premolar autotransplantation has recently been proposed as a viable option for managing ankylosed incisors [18]. Nonetheless, in the present case, this approach is contraindicated due to the absence of an extraction indication for the premolars. Moreover, this procedure entails inherent risks, with the transplants remaining prone to replacement resorption and ankylosis [19]. Considering the respective merits and drawbacks of all available therapeutic modalities, surgical luxation followed by orthodontic repositioning was determined to be the most appropriate intervention for this patient.

It has been suggested that surgical repositioning is recommended for severely traumatized teeth that do not respond to orthodontic force, typically involving immediate repositioning under local anesthesia [20]. However, in the present case, surgical luxation combined with orthodontic traction was adopted instead of immediate surgical repositioning, and a satisfactory clinical outcome was achieved. Such an approach has been previously described and may provide an additional therapeutic option in selected clinical scenarios. Nonetheless, it should be interpreted with caution and not be regarded as a substitute for established guideline‐based recommendations. Surgical luxation is theoretically grounded in the concept that sufficient disruption of the ankylotic interface, while preserving periapical blood supply, may elicit an inflammatory response conducive to the regeneration of a functional periodontal ligament [9]. Regarding the timing of orthodontic force application, a previous study advocates immediate postluxation activation to prevent reankylosis [21]. However, reankylosis may still occur despite timely orthodontic intervention. In this patient, immediate application of orthodontic force following surgical luxation of Tooth 21 resulted in a prompt and clinically significant extrusion within one month. Fortunately, no evidence of reankylosis was detected during the two‐year posttreatment follow‐up.

A recent systematic review and meta‐analysis demonstrated that spontaneous re‐eruption is associated with a significantly lower risk of marginal bone defects compared to both surgical and orthodontic repositioning approaches [5]. These findings suggest that active repositioning may predispose to marginal bone compromise, potentially affecting long‐term periodontal stability and esthetic outcomes. Consistent with these observations, orthodontic extrusion in the present case failed to promote coronal migration of the marginal bone, and the bone defect resulting from the initial traumatic intrusion persisted unchanged even after two years of posttreatment follow‐up. This underscores a critical clinical implication: orthodontic repositioning alone may be insufficient to achieve optimal esthetic outcomes in the anterior region. A more integrated approach, incorporating adjunctive procedures such as localized bone grafting or mucogingival surgery, may be warranted to facilitate vertical bone regeneration and support favorable soft tissue contours.

Despite achieving the primary therapeutic goal of managing the ankylosed incisor, certain limitations of the orthodontic phase warrant consideration. The patient’s guardians elected not to pursue comprehensive orthodontic treatment, and no posttreatment retention protocol was implemented. These factors may have contributed to residual discrepancies in tooth alignment and occlusal relationships at treatment completion, as well as the minor relapse observed during the two‐year follow‐up period. In retrospect, the use of a bonded flexible spiral retainer following appliance removal might have improved long‐term stability. Furthermore, several adjunctive measures might have further optimized the treatment outcome. During the orthodontic repositioning phase, a piggyback archwire system used in conjunction with sequential archwire progression may have provided a more continuous and controlled extrusive force for Tooth 21. Such a system may also enhance anchorage control by stabilizing the adjacent teeth and minimizing unwanted tooth movement resulting from reciprocal orthodontic forces. Esthetically, multiple residual interdental black triangles persisted in the maxillary anterior region at treatment completion. Additional interdisciplinary management, including restorative, periodontal, or orthodontic approaches aimed at reducing open gingival embrasures, might have further improved the final esthetic outcome.

Given the extent of the initial trauma, the patient remains under vigilant long‐term follow‐up, as the risk of late complications cannot yet be fully excluded. While the short‐term clinical outcome has been favorable, definitive treatment planning is ongoing. In particular, future prosthetic rehabilitation to address the defected teeth in adulthood must be carefully anticipated and integrated into the overall management.

## 4. Conclusions

Severe intrusive luxation is associated with a high risk of complications, including ankylosis. Careful and continuous follow‐up after dental trauma is essential for early detection of adverse outcomes such as arrested eruption and ankylosis.

Management of intrusive luxation should be guided by the evidence‐based recommendations of the IADT. For mature permanent teeth with an intrusion depth exceeding 3 mm, immediate active repositioning—either orthodontic or surgical—is recommended. In the present case, an initial period of observation was undertaken as part of an individualized treatment approach; however, this should be interpreted within the specific clinical context and not as a general recommendation for similar cases.

Once ankylosis is established, orthodontic traction alone is typically ineffective. In such situations, surgical luxation combined with orthodontic traction may facilitate tooth repositioning. Nevertheless, this approach has limited ability to restore marginal bone defects resulting from intrusive injury, which remains a significant challenge in long‐term management.

## Author Contributions

K.L. contributed to the conception and design of the work and drafted the manuscript. M.T. contributed to the conception and design of the work and completed the treatment. D.Y. contributed to the design of the work and revised the manuscript. M.C. contributed to the acquisition of data and revised the manuscript.

## Funding

No funding was received for this work.

## Disclosure

All authors read and approved the final manuscript.

## Ethics Statement

The study was approved by the Research Ethics Committee of Yantai Stomatological Hospital. The written consent was obtained from the patient’s parents.

## Consent

Please see the Ethics Statement.

## Conflicts of Interest

The authors declare no conflicts of interest.

## Data Availability

The data and materials that support the findings of this study are available from the corresponding author upon reasonable request.
